# Hospital at night: an organizational design that provides safer care at night

**DOI:** 10.1186/1472-6920-14-S1-S17

**Published:** 2014-12-11

**Authors:** Diana Hamilton-Fairley, John Coakley, Fiona Moss

**Affiliations:** 1 Guy’s and St Thomas’ NHS Foundation Trust, London, UK; 2 Homerton University Hospital, NHS Foundation Trust, London, UK; 3 London Deanery, London, UK

## Abstract

The reduction in the working hours of doctors represents a challenge to the delivery of medical care to acutely sick patients 24 hours a day. Increasing the number of doctors to support multiple specialty rosters is not the solution for economic or organizational reasons. This paper outlines an alternative, economically viable multidisciplinary solution that has been shown to improve patient outcomes and provides organizational consistency. The change requires strong clinical leadership, with organizational commitment to both cultural and structural change. Careful attention to ensuring the teams possess the appropriate competencies, implementing a reliable process to identify the sickest patients and escalate their care, and structuring rotas efficiently are essential features of success.

## Introduction

“Medicine used to be simple, ineffective, and relatively safe. Now it is complex, effective, and potentially dangerous [1].”

The quality of the working lives of junior doctors in the United Kingdom has been a matter of media and public concern for many years [2]. In 1993, most doctors in the country were working over 84 hours per week, and some in acute admitting specialties were working more than 100 hours. Shift lengths varied from 32 hours to over 90 hours with no guaranteed breaks [3]. Now, under the European Working Time Regulation (EWTR), British doctors-in-training work an average (over 26 weeks) of 48 hours per week. Reductions in the working hours of doctors-in-training have been challenging to implement, and continue to be an emotionally charged issue. Concerns expressed publicly and privately in medical and health care circles include the negative impact on patients of a perceived reduction in continuity of care. The “craft” specialities question the feasibility of training doctors to become competent independent practitioners in a shorter working week. Reducing the hours worked from 84 to 48 per week has contributed to an estimated reduction in total hours worked over a postgraduate (residency) surgical training program from 30,000 to 8,000 [4].

The task of reducing doctors’ working hours should be considered against the background of continuing significant advances in medical technology and changes in the delivery of health care. Twenty-first century interventions are much more effective than those available in the 1980s; much more care is delivered in ambulatory settings or requires a shorter time in hospital. Working patterns must also be based on knowledge of research about the impact of working at night on practitioners’ health and patient safety. In this paper we briefly outline some of the evidence that should inform decisions about doctors’ working patterns and how to support safe care for patients during the night. We then describe the experience of two hospitals – both part of the UK Hospital at Night (H@N)/Taking Care 24/7 project – that have taken a “whole systems” approach to organizing care at night [5]. The experiences of these hospitals show that it is possible to reduce hours while providing good training and improving patient safety.

## Background

### Impact of working long hours and working at night

The impact of physician fatigue on patient safety should be the principal focus of this discussion. The *number* of hours worked per week or month is less important than the *impact* of working long hours and, in particular, of working at night. At night, complex tasks become more difficult to execute, and the acuity of decision making can be blunted. Medical errors, adverse events, and attention failure have been noted in interns working extended shifts (those greater than 24 hours) [6].

A systematic review of the impact of working long hours (more than 48 per week) and shift work revealed adverse effects on the worker’s health as well as on his or her family and social life [7]. Other reported effects include an association between road traffic accidents and shifts longer than 24 hours; this risk increases with the frequency of these extended shifts [8].With this evidence in mind, efforts should be concentrated on minimizing the number of people involved in providing care at night, reducing the number of night shifts per individual, and reducing the total number of hours worked.

### Doctors in training and patient safety

Evidence suggests that patients fare less well at night when they are cared for by doctors in training programs. The UK National Confidential Enquiries into Perioperative Deaths (CEPOD and NCEPOD), which began in 1982, found an association between perioperative death and being operated on by a junior surgeon out of “normal working hours” [9]. In light of these national analyses, UK hospitals set up daytime “CEPOD” theatre lists to ensure that the majority of emergency operations were done during normal working hours. More recently, 8% of trainees who responded to the General Medical Council annual trainee survey reported that they had made a serious or potentially serious error in the last month; a substantial proportion of trainees attributed these errors to sleep deprivation and working longer hours [10].

### Mitigating the effects of working at night

Working patterns should reflect an approach that is safest for patients and healthiest for doctors and other staff. Although shift work can harm health, this harm can be mitigated. Fatigue, but not necessarily performance, is worse on longer shifts. Shift schedules designed by workers encourage good performance, although many workers favour longer shifts to gain longer rest periods. Frequent 24-hour shifts (more than 1:3 days) have been shown to increase the risk of serious medical errors [7,11].

Most research into minimizing the deleterious effects of shift work has concentrated on rotating three eight-hour shifts. Night shifts are the most harmful to health; therefore, night work should be reduced as much as possible. The rapid rotation of shifts (a change every few days) is preferable to a slow rotation, as the former interferes less with the circadian rhythms. As well, clockwise rotation (morning, afternoon, night) is preferable to counter-clockwise rotation in an eight-hour shift model. Twelve-hour shift models with quick changeovers (e.g., the morning and the night shift in the same 24-hour period) should be avoided to allow for longer rest periods between shifts. Finally, a later start for the morning shift reduces the truncation of the previous sleep period, particularly for REM sleep. Factors that can ameliorate shift work include workplace improvements in catering, supervision, health care, transportation, and recreational facilities. Some limited evidence also suggests that bright light on the night shift might offset some of the circadian effects of day–night changes [5].

### The impact of daytime system inefficiencies on nighttime work

**Figure 1:**
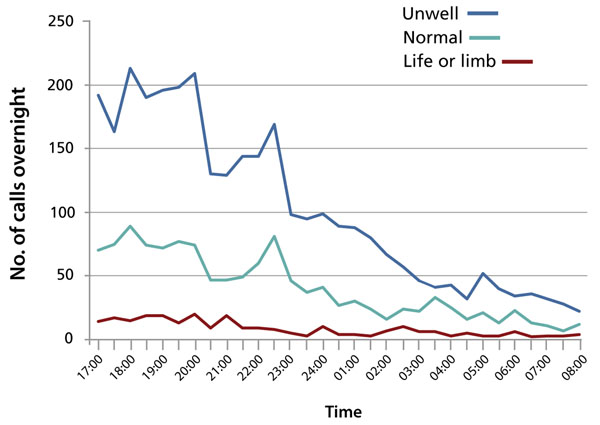
Average number of calls received per overnight shift. Data source: UK National Audit 2003.

Understanding the nature of clinical work done at night is key to developing organizational structures that support safe patient care. Many of the tasks that doctors undertake “out of hours” have been shown to be routine, “inappropriate,” or unrelated to acute need [5]. Although a certain proportion of patients will require expert and sometimes life-saving medical attention at night, an analysis of the competencies required for out-of-hours care of inpatients found that the need for specialist intervention was rare; most calls required doctors with generic competencies in all specialties (from orthopaedics to oncology). On average, only five skills (e.g., management of acute coronary syndrome, compartment syndrome) were identified as being specialty-specific (unpublished data). Nursing and allied health professionals already perform many generic tasks, and other tasks can readily be performed by practitioners in extended-practice or alternative health care roles (e.g., physician assistants). System inefficiencies prevent the completion of routine daytime tasks, which are rolled over to the night, and so most nighttime calls prove not to be of a “life-and-limb” nature (Figure 1) [5].

### Night teams for modern care

Although changes in medical technology over the past two decades have brought about significant improvements in the effectiveness of care, hospitals still require working patterns that enable the safe delivery of care while minimizing negative effects on the health of care providers. Two fundamental points about delivering safe patient care at night have arisen from the research regarding working at night: (1) tasks done at night should be minimized, such that as much work as possible is done during the “normal” day and (2) only competent practitioners should undertake complex tasks during night hours. Working patterns must also allow doctors and other health care professionals to be trained in a manner that does not put patients at risk. From the evidence available, it is possible to derive principles for designing team approaches for delivering safe nighttime care. These principles include the following:

• Those expected to contribute to out-of-hours work should be involved in the rota design while ensuring that the duration of shifts and the hours worked each week meet the agreed standards.

• Those on-call at night should be asked to do night work for three or four nights at a time, as this has less impact on health and is safer for patients than either working either weeks of nights or single nights.

• Those on-call at night should be competent to undertake the predicted work.

• Those on-call at night should work as members of a team with clearly defined roles and a designated leader.

• Those working at night should be encouraged to eat well-balanced meals and avoid caffeine.

• Provision should be made for those working at night to take short naps in a quiet environment**.**

• Night teams must be multi-professional so that they have the necessary skill mix.

### The Hospital at Night project

In the United Kingdom, the pressure to reduce junior doctors’ hours prompted a national project sponsored by the Department of Health: Hospital at Night (H@N). The H@N project gathered evidence and provided guidance for the development of safe and functioning teams to care for patients at night. The project has moved on from H@N to Hospital 24/7, as it is clear that good team functioning is critical for delivering high-quality and safe care both during the day and at night [5].

Changing to H@N from a standard “firm” and “on-call-rota” approach to night care requires significant organizational and cultural adaptation. We report here the experience of two London hospitals – Guy’s and St Thomas’ NHS Foundation Trust, a teaching hospital, and Homerton University Hospital NHS Foundation Trust, a district general hospital – that have successfully implemented H@N and Hospital 24/7. These case studies illustrate not only the degree of organizational change required, but also the benefits of this new approach. A comparison of the changes made at these two sites is outlined in Table 1.

**Table 1:** Comparison of changes at Guy’s and St Thomas’ NHS Foundation Trust versus Homerton University Hospital NHS Foundation Trust

	Guy’s and St Thomas’ NHS Foundation Trust	Homerton University Hospital NHS Foundation Trust
Key drivers	Patient safety Regulatory requirement to reduce doctors’ hours Controlling costs Maintaining/improving medical training	Patient safety Regulatory requirement to reduce doctors’ hours Maintaining/improving medical training

Key outcomes	Reduction in HSMR Reduction in serious incidents Reduction in health care–associated infections 100% compliance with EWTR Maximum cost of £2.4 million	Reduction in HSMR No increase in serious incidents Reduction in health care–associated infections 100% compliance with EWTR

Data collection	Analysis of on-call duties Analysis of rotas Creation of competency matrix	Presentations and discussions on how to improve patient care

Hospital at Night	SNPs with both clinical and site management responsibilities Structured handover at the same time for all specialties Baton bleeper for face-to-face handovers First point of contact for wards and other areas Twilight shifts for specialties (majority removed for overnight) On-call teams covering patients from all specialties Consultant ward rounds by Surgery and General Internal Medicine every 12 hours for all admissions SNPs see, assess, treat, and /or refer acutely ill patients 18 pathways (with associated protocols) for common emergencies	Clinical Site Manager Team with both clinical and site management responsibilities First point of contact for wards and other areas Single team for emergency admissions via emergency department Single team to cover inpatients

Taking Care 24/7	Extension of H@N into the day Physician of the week for surgical inpatients working with surgical teams Single escalation system for both sites Single admissions area for elective surgical patients Handovers for planned discharge and weekend care Regular contact with wards and doctors by SNP every 6 hours 24/7	Separation of elective and emergency work Single admissions area for elective work Acute Care Unit Doctors work only in one or the other pathway for set periods of time, thereby maximizing training opportunities Consultant in General Internal Medicine present in emergency area 12 hours per day Factual handover at 8 a.m. Elective to emergency team handover at 4 p.m.

Impact on patient care	Initial reduction in HSMR Sustained reduction in serious incidents Reduction in health care–associated infections Reduction in in-hospital cardiac arrests Reduction in lengths of stay	Initial reduction in HSMR Reduction in health care–associated infections

Financial impact	H@N: £4.1 million saving; £2.4 million in recurrent costs 24/7: closure of 250 beds	H@N: £100,000 saving 24/7: £600,000 saving; £250,000 in recurrent costs

Educational impact	H@N: no change in feedback from junior doctors 24/7: improved teaching time participation for most junior staff and physicians (daily seminar from physician of the week)	H@N: no change in feedback from junior doctors Sustained hours of direct supervision/elective work Reduction in hours spent in “acute care team” for each doctor Educational handover at 10 a.m.

Lessons learned	Need involvement of all staff, not just medical Need good, relevant data Training for staff who are extending/changing their role The change is part of a whole system change that continues to evolve; 24/7 is only one part that contributes to the improvement as a whole	Need involvement of all staff, not just medical Need good, relevant data Training for staff who are extending/changing their role The change is part of a whole system change that continues to evolve; 24/7 is only one part that contributes to the improvement as a whole

Sustainability	Yes – no appetite to return to the on-call system	Yes – no appetite to return to the on-call system

EWTR = European Working Time Regulation; HSMR = Hospital Standardised Mortality Ratio; SNP = senior nurse practitioner

## Case 1: Guy’s and St Thomas’ NHS Foundation Trust

### Context

Guy’s and St Thomas’ NHS Foundation Trust is a large secondary and tertiary teaching hospital with an annual budget of £1 billion. It serves a deprived local population of 1.3 million, providing regional and national referral services in most major specialties and subspecialties. For example, this hospital is the specialist cancer centre for South East London (with a population of 2.6 million) and the children’s referral centre for South East England (with a population of 3.5 million). This hospital operates at two sites:

• Guy’s – predominantly an elective surgery and cancer centre with a total of 350 beds

• St Thomas’ – an acute-care site with 850 beds as well as the 120-bed Evelina Children’s Hospital

The Guy’s and St Thomas’ NHS Foundation Trust has approximately one million patient contacts per year. Each year there are approximately 120,000 visits to the adult emergency department and 30,000 to the children’s emergency department. In addition, the hospital delivers approximately 6,800 babies each year.

The hospital employs over 11,000 staff, including 1,970 doctors, of which 870 are consultants and 720 are doctors in postgraduate training (all grades of residents) and 380 in non-training grades.

### Outline of problem

In 2003 there were a total of 144 on-call rotas. These medical teams – consisting of two levels of doctors on-call at any one time, with a consultant on-call from home – were on-call only for their own patients. The work intensity experienced by these teams varied according to specialty. Emergency and admitting, General Internal Medicine, Obstetrics, Anaesthetics, Intensive/High Dependency Care, and Pediatrics teams tended to work hard throughout the day and night. In contrast, general ward patients tended to require medical attention only until 8:30 p.m., and most other specialty physicians slept for at least five hours per night without being disturbed.

The junior member of the medical team was typically the first to be called to attend to a patient. Senior doctors were usually called later and only if the junior doctor thought it was necessary. It is well documented that this was often the root cause of a poor outcome [9].

The hospital’s risk record in 2002 included 16 serious adverse incidents that were considered to be avoidable. There were also a significant number of cases of methicillin-resistant *Staphylococcus aureus* (MRSA) (81 per year). In addition, the Hospital Standardised Mortality Ratio (HSMR) was at the United Kingdom average.

Over a six-month period ending in August 2004, the hospital started to reduce junior doctors’ working hours to an average of 48 per week to comply with the EWTR. As the hospital’s first priority was patient safety, any proposed solution had to improve patient care.

### Key measures of improvement

The vision was to reorganize care so that all patients had access to the right person with the right skills for their needs at the right time. Patient care and patient safety were the prime goals, along with the achievement of the EWTR. Identified outcome measures included a reduction in serious adverse incidents, a reduction in the number of cases of MRSA, and a reduction in the HSMR.

### Process of gathering information

Colleagues in Human Resources collected information about all medical rotas. A financial costing was done of maintaining on-call rotas. In addition, an analysis of on-call work from 5 p.m. to 8 a.m. was undertaken. Meetings to understand patients requirements out of hours were held with all staff, including porters, transport, medical records, and IT. This allowed us to identify all required competencies in addition to clinical competencies, such as the ability to access databases and medical records, equipment such as wheelchairs, and services such as radiology, the Intensive Therapy Unit, High-Dependency Care, and the morturary.

### Strategy for change

**Figure 2:**
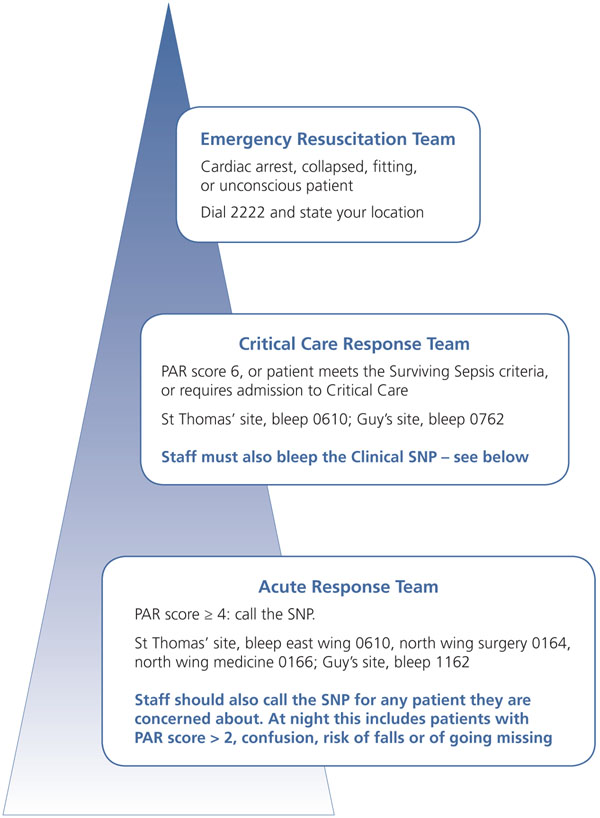
Escalation of care for sick patients at Guy’s and St. Thomas’ NHS Foundation Trust. SNP = senior nurse practitioner; PAR = patient at risk.

**Figure 3:**
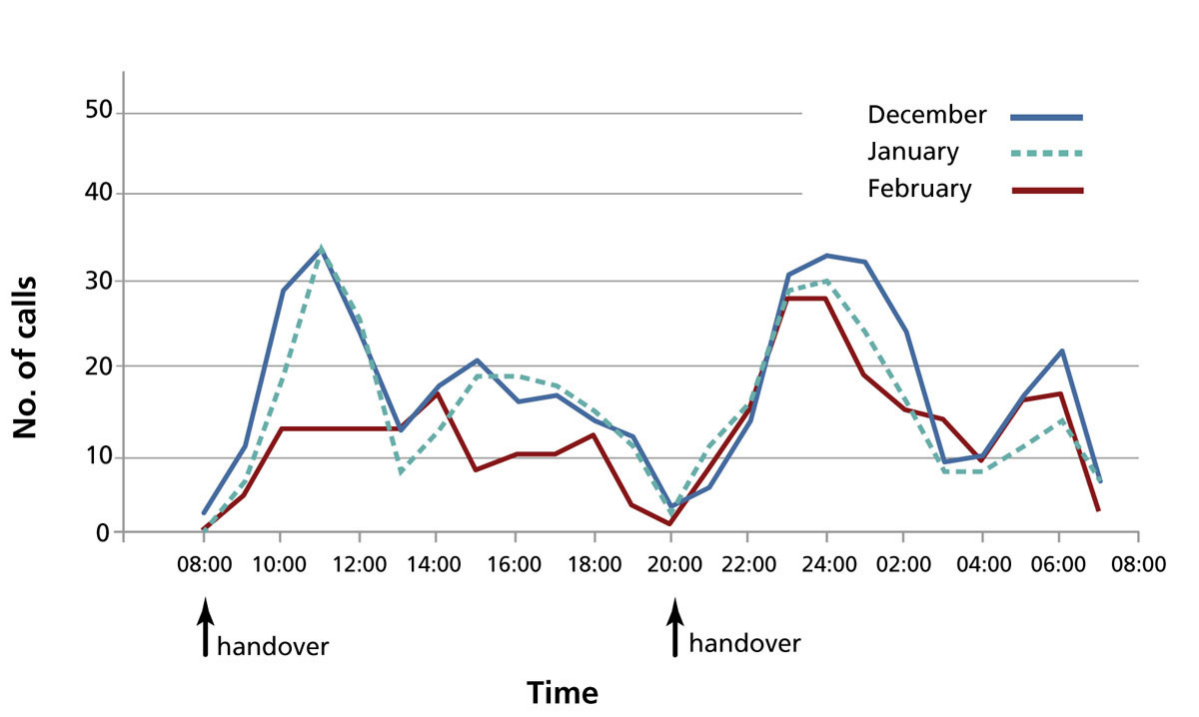
Average total calls received per day at Guy’s Hospital, London, UK, after the implementation of Taking Care 24/7, 2008–2009. Data source: Senior Nurse Practitioners, Guy’s and St Thomas’ Foundation Trust.

A review of all data showed that better care could be provided on-call by fewer people with the introduction of three multi-professional teams across the hospital sites, with a fourth for the Evelina Children’s Hospital. In addition, only minimal changes were made to the emergency admission teams. The strategy was operationalized as follows:

• Each team is led by a senior nurse practitioner (SNP) who takes all calls from all areas. This strategy of using SNPs as leaders provides consistency because these individuals are permanent staff who have a thorough knowledge of their hospital and ward staff, and they are able to provide effective site management, including bed management.

• All patients are given a score (Patient at Risk [PAR]) at each point of observations, and any changes in their scores are reported to the SNP who then assesses the patient, determines his or her priority, starts treatment according to agreed-upon protocols, and refers the patient to the relevant doctor, physiotherapist, nurse, etc.

• There is an agreed-upon escalation policy that involves, as necessary, referral to the Medical Emergency Team (critical care outreach). (See Figure 2 for additional details.)

• Handover was synchronized across the Trusts, and a 30-minute period was built into shift times for handover. (See Figure 3 for more details.)

• Each team holds a “baton” bleeper that, at the end of shift, has to be handed over. This ensures that there is a face-to-face handover.

• Specialist teams (referred to as twilight teams) stay on-site until 9 p.m. (with handover at 8.30 p.m.) to complete all routine tasks and ensure that all their patients are stable with no ongoing needs.

• A representative from each twilight team attends handover to report on all patients requiring input/additional observation through the night.

• The overnight non-acute rotas were reduced to a minimum. As well, junior surgical rotas were merged and staff were shared.

• Some specialties (e.g., Obstetrics, Anaesthesia, Neonatology, Intensive Care) could not be included because of the requirement for specialty-specific competencies throughout the 24 hours.

• Team Standard Operational Procedures were developed. These included roles, competency checklists, handover, escalation policies for acutely sick patients, and agreed-upon protocols and guidelines for the most common conditions occurring overnight. Nurses were trained to prescribe against agreed-upon protocols for commonly occurring conditions (e.g., oliguria, asthma, cardiac failure, pain relief). Competencies were checked at induction on starting work in the Trust and confirmed with educational supervisors from the doctor’s portfolio of training/log book.

£2.4 million was available for this project. The resources were used to pay for the following:

• A clinical champion for one day per week who worked with a project manager (full-time).

• Human Resources time to analyze and help redesign rotas with input from clinicians and junior doctors.

• Training for the SNPs in advanced clinical skills, including full patient assessment (particularly cardiorespiratory and neurological systems, interpreting electrocardiograms, chest X-rays, and performing and interpreting arterial blood gases).

• Increasing the number of doctors on the on-call roster for emergency surgery from six to nine to achieve compliance.

• Increasing the number of SNPs.

• Funding for advanced nurse practitioners in some areas (e.g., Emergency, Gynecology, Ophthalmology) to reduce the amount of work requiring doctor input.

• Training of all ward staff to calculate accurate PAR scores, with a weekly audit to ensure this was being done correctly.

• Training on induction of the surgical trainees so that they could cross cover urological and ear, nose, and throat emergencies.

### Effects of change on patients

The analysis of benefits of the night teams – which had been first introduced in 2004 – took place in 2009. Overall, analysis showed that patients are seen in a timely fashion and that more than 95% of the time routine tasks are completed before the night shift starts. More importantly, we found only one serious adverse incident in 2007; this was maintained to less than three per annum up to 2011. There was also a reduction in HSMR to 0.87 in first year of implementation; the hospital has remained in the top three in London since that time. In addition, there was a reduction in MRSA cases from 81 per annum to 7 per annum.

### Effects of change on education

On the night team, most junior doctors work alongside, and are supported and supervised by, SNPs who are trained to do procedural assessments. Under this system, the junior doctors’ education continues overnight. More senior medical staff are always available for complex cases, and junior staff are never asked to perform beyond their competence. Reports from junior staff include high praise for the SNPs, the handover is considered effective and educational, and those in Foundation years (the first two years post-qualification) have requested to do more night shifts. More senior surgeons (more than five years post-qualification) are on-site until 11 p.m., as no surgical procedures are done at night unless they are considered to be life-saving. This has minimized the effect on training during daytime hours. Consultants are called in a little more frequently; however, this is always considered to be appropriate (e.g., for life-saving surgery).

### Effects of change on finances

The estimated cost to maintain the original 144 on-call rotas in 2003 was £7 million. As described, it cost £2.4 million to introduce the changes. The number of rotas was reduced to 102, of which only 25 are physically present on-site. The remainder go home at 9 p.m. Only 15 remain on-duty from home, responding by phone more than 80% of the time; however, they are expected to attend within 30 minutes if required. Consultants are also on-call from home. This system reduced the recurrent costs from £7 million per annum to £2.6 million (in 2009 dollars).

Other changes introduced at the same time included the following:

• Extending this system into the day.

• Developing a medical perioperative support team for the elderly.

• Naming a physician for each week to oversee the elective surgical and oncology patients; this has reduced the in-patient length of stay and reduced the number of in-hospital cardiac arrests from an average of 13 per month to 3 per month for the nine months between March and December 2011 (with no in-hospital cardiac arrests for 102 consecutive days).

Since 2004, shortening the length of stay has led to a reduction in the bed base by 250 beds, despite an overall increase in in-patient episodes of 13% (average of 3% per annum). This reduction in the bed base has saved £1 million per annum per 25 beds closed and has increased the income generated as a result of the increased number of Finished Consultant Episodes (the basis on which NHS acute trusts are paid).

### Lessons learned

This major cultural change took time both to start and to embed. The main hurdle was for specialists to fully trust that the overnight urgent care needs of their patients were mostly generic and that they very rarely required specialist intervention. The impact on the hospital has been more far-reaching than we had imagined. What began as H@N has enabled the introduction of many other organizational changes that have improved care 24/7.

### Message for others

It is key to have a senior clinical champion who believes that this change will improve patient care. It is also important to do the following: collect initial data; engage in detailed discussions and ensure that there is sufficient time to engage with all groups of staff; train those individuals whose roles are changing; and focus on the quality and safety of care as well as on the number of hours worked. The cultural change is perhaps greatest for doctors, but nurses had to be convinced that they are the most appropriate professional to lead the teams and that they have the capability to enhance their roles.

### Next steps

The day and night systems are now the same. There is now a single point of contact, available at one number 24/7, for urgent care. SNPs work proactively by “rounding” all wards every six hours. All patients who are stepped down from level 3 (Intensive Care) and 2 (High Dependency Care) are reviewed every 4 hours by the SNP for the first 12 hours; this is in addition to increased observation times by the nurses on the wards. Surgeons can now spend longer in theatre, as physicians are on duty for post-operative medical care. Residents and other doctors-in-training report that they are being supported and that they receive good training and teaching.

## Case 2: Homerton University Hospital NHS Foundation Trust

### Context

Homerton University Hospital is a 550-bed district general hospital located in Hackney, an inner city area with a multi-ethnic, deprived population of approximately 250,000 to 300,000 people. The hospital provides acute hospital and community services for the local population. It also offers some tertiary services, including bariatric surgery, neurological rehabilitation, neonatal intensive care, and high-risk maternity care.

On average there are 120,000 emergency department visits per year. In addition, the hospital delivers 5,000 babies each year. The annual turnover is in excess of £230 million.

### Outline of problem (patient-centred)

There was a recognition in 2003 that significant and robust service redesign was the only way to guarantee good quality care and patient safety day and night while also providing adequate training opportunities, controlling costs, and meeting the EWTR (i.e., 48 hours per week with a maximum shift length of 13 hours) without increasing the number of doctors required.

### Process of gathering information

In 2003 we monitored all junior doctors’ activity during their night shift, in some cases by using diaries and in others by shadowing them overnight.

This exercise revealed the following:

• Activity was variable across grades and specialties from minimal to extremely busy.

• Some work should have been completed during the day or could have waited until the next day.

• Some work did not need to be done by doctors.

• “Life-and-limb-threatening” work was relatively rare, and sometimes it should have been done by more senior medical staff.

• There was evidence of poor systematic handover of patients.

### Strategy for change

We introduced H@N in 2003 with a Clinical Site Manager Team. In August 2007, two years ahead of the 2009 deadline, we took this to the next stage: “Taking Care 24/7,” with a maximum 48-hour week for all doctors.

#### Stage 1 – Hospital at Night (2003)

Clinical Site Managers – highly trained nurses usually with a background in acute/emergency/critical care medicine – were introduced to coordinate the night work and to be the first on-call. These managers screened out tasks that did not need to be done by a doctor and ensured that the most-appropriate doctor, one who was often not the most junior, was called to perform any required tasks. Having this one point of initial contact also helped to prevent multiple calls being made to different doctors for the same task. These individuals also supervised – and ensured maximum attendance at – the hospital-wide nighttime handover. In addition, an analysis of work performed by resident on-call junior doctors showed that many of them had little to do overnight. Accordingly, these doctors were removed from the on-call rotas.

Some senior doctors were concerned that these changes were driven by agendas other than patient care (e.g., to save costs or to make consultants work more hours), and this was partly true. To help alleviate these concerns, a series of presentations and discussions were held for all grades of medical staff, other clinicians, and managers to describe how patient care could be improved with the new approach. In a short amount of time the value of the Clinical Site Manager Team as first point of contact for out-of-hours care was recognized and welcomed.

#### Stage 2 – Taking Care 24/7 (2007)

Staff were divided into elective (“cold”) and emergency (“hot”) teams. Trainee doctors spent time, usually periods of weeks, on each team, and were thus able to focus exclusively on either “hot” or “cold” work for both service delivery and training purposes at different times.

Following a review of patient flows prior to implementing Taking Care 24/7, all elective surgical admissions were directed to a single ward area. From there, they went to theatres and, finally, to their in-patient recovery ward. This reduced congestion on receiving wards, as patients scheduled for discharge could stay until mid-morning, and also prevent the unnecessary displacement of newly admitted patients. All emergency admissions (except maternity and children) were admitted to the Acute Care Unit, which was staffed by the Acute Care Team.

Importantly, between 2003 and 2007 we had already changed the working patterns of consultants so that they were required to provide senior cover for an extended 12-hour working day for emergency care on weekdays, with a slightly shorter time commitment on weekends.

As Taking Care 24/7 evolved, it became obvious that we had made a number of errors that had to be corrected. For instance, handovers were often unfocused and lengthy, and they were occurring too frequently. To remedy this situation, we changed handovers to a brief factual handover at 8 a.m., a more contemplative and educational handover after the post take ward round at 10:30 a.m., and a cold to hot team handover at 4 p.m. The night handover continued as it had as part of H@N.

### Resources needed

For both service redesign projects we employed a project manager for a little over 12 months. We used no other additional resources, although most staff input took place during normal working hours and, therefore, theoretical costs were clearly involved.

### Effects of change on patient care

**Figure 4:**
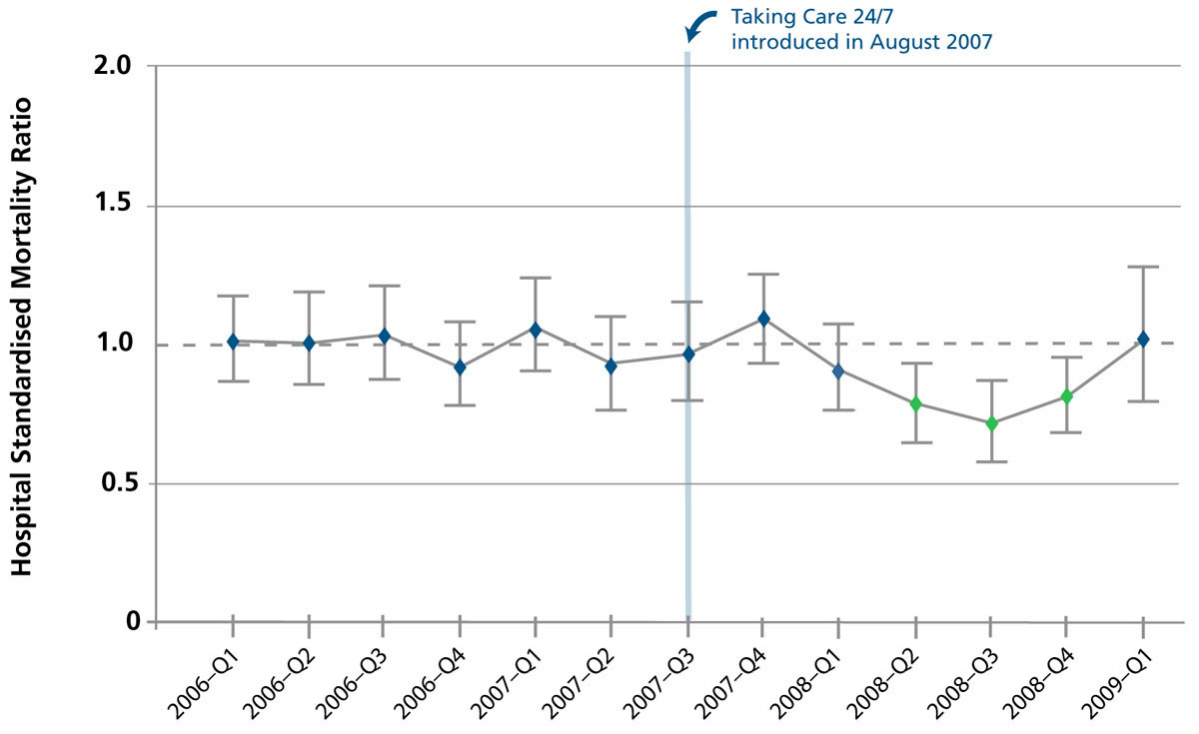
Quarterly in-hospital relative risk of mortality, with 95% confidence intervals, for all non-elective admissions, Homerton University Hospital, London, UK. Values shown in green are statistically significant deviations from expected values.

We looked at a number of indicators to assess the impact of these changes on patient care. The HSMR fell shortly after we introduced Taking Care 24/7, before gradually returning to baseline (Figure 4). It is important to emphasize that improvements to patient care cannot be attributed solely to the changes in the junior doctors’ hours of work. The way services were delivered changed, in particular the introduction of an extended working day for senior doctors. In addition, the elective and emergency admissions were each placed in a specific location with dedicated medical and nursing teams specific to each group with no possibility for split or confused duties for each group of staff. Other, less quantifiable, things inevitably changed at the same time. Nevertheless, we implemented what had been regarded, by some, as dangerous reforms to junior doctors’ hours without detriment to patient safety. The following points, in particular, were observed:

• There were no deleterious effects on length of stay or the number of admissions and readmissions.

• No significant untoward incidents were reported at the time of implementation or since that are attributable to the changes.

• The incidence of health care–associated infections fell – and continues to fall – after the implementation of Taking Care 24/7.

### Effects of change on education

We were able to maintain hours of direct supervision of trainees by consultants in their elective time because during that time there was no night on-call or weekend work. Taken over a whole year, the time devoted to cold shifts was maintained, while that devoted to hot shifts was reduced.

### Effects of change on finances

The changes associated with H@N saved approximately £100,000 per year (in 2003 dollars). The savings associated with Taking Care 24/7 were approximately £600,000. For the latter, additional costs incurred were £250,000 (in 2007 dollars) to pay for the increased nursing presence and some junior staff numbers. Both of these sums were recurrent. Had we not radically changed the service model, the additional costs would have been very much greater and no savings would have been realized.

The savings were achieved by reducing all doctors’ hours of work to 48 per week and removing some from resident on-call work completely.

### Lessons learned

The key to successful implementation of these changes was to involve those responsible for delivering the changes in the planning process and to manage the changes in clear phases. We used evidence from the first phase to build up confidence; this allowed us to progress to the further phases of implementation. It was important for both system changes to encourage direct and indirect feedback so that problems could be dealt with and successes celebrated as soon as possible.

### Message for others

Effective communication is vital to implementing change of this magnitude. Some of the better suggestions for improvement to the system were made by doctors in training, those who were living through these changes to the way they worked. Feedback, even hostile, must be encouraged and actively sought.

It is essential to focus on the demands of the service and the training needs, not on the hours per se. Dealing with the hours of work in isolation – or even as the prime mover – will not provide solutions, but will instead exacerbate problems and fault lines in the current system.

### Current approach

We plan to continue with the service redesign. It is becoming clear that a senior consultant presence is required seven days a week and even, possibly, around the clock for acute care. This currently presents major financial challenges.

## Discussion

Working excessive hours puts patients at unacceptable risk, is bad for the health of practitioners, and increases the risk of both accidents in the workplace and car accidents after leaving work. Health care in general – and medicine in particular – has been slow to develop ways of working that enable safe delivery of modern medical care within a safe environment for the staff who are delivering that care. Changes to working hours have perceived, but not proven, negative effects on continuity of care and the training of junior doctors. The case studies we describe outline how it is possible to organize health care staff so that the twin aims of safety for patients and staff and a good environment for training can be realized. While the principles might be the same, it is clear that different hospitals will take somewhat different approaches to the task of maintaining sufficient and appropriate medical cover for patients being admitted as emergency or elective patients. An overview of the systematic approach described in these two hospitals is summarized in Table 1.

The extended role of nurses in both of these models is striking, in particular their role as coordinator for the hospital site and for the clinical assessment of deteriorating patients. The availability of, and appropriate training for, these staff members is crucial to the success of the system. Initial fears that this would erode the role of the doctor have not been borne out as this system tends to allow doctors to be called appropriately to optimize the management of patient care.

The coordination of all admissions and discharges by a single person who is aware of the organization of the whole hospital is key to the success of separating the elective and acute pathways for patients. This also allows teams to concentrate on one pathway without worrying about the other. This appears to improve patient care through improved teamwork and increased efficiency in moving through the elements of the pathway facilitated by a single team. Concentrating on the way of working and minimizing the opportunity for medical errors (reducing fatigue through proper rest and minimizing disruption to the circadian rhythm) also appears to minimize costs and can lead to sustained financial savings.

Work on competencies required for overnight care has allowed doctors with generic skills to be available for overnight emergencies for a group of specialties (e.g., for surgical patients, junior residents can cover inpatients from all surgical specialties with distant support from senior residents for the rare specialty-specific emergency).

Senior residents can be on-call from home, taking referrals from the H@N team or other centres. A single team was needed to cover admissions for the emergency department. Thus fewer doctors are present in the hospital, although they work harder in short bursts. The key to the success of such a system is that all clinical professionals work together with an organized coordinated approach that includes assessment, prioritization, treatment, and, if appropriate, further referral. The team leader, usually a senior advanced nurse practitioner, receives all calls and coordinates all activity within the hospital.

The important messages are that changing from a standard approach to doctors’ duty hours to a coordinated H@N or Hospital 24/7 approach requires significant organizational change that will have an impact on the working practices of many clinicians. This change is not easy, and it requires considerable determination and continuing input from senior staff to embed and maintain it. The difficulty lies in persuading many dedicated individuals that working differently will indeed provide better and safer care. The hospitals in the United Kingdom that have implemented this change have all shown improvement in patient safety, with some evidence of reduced costs overall.

## Competing interests

The authors declare that they have no competing interests.

## Declarations

Resources and secretariat support for this project was provided by the Royal College.

This article has been published as part of *BMC Medical Education* Volume 14 Supplement 1, 2014: Resident duty hours across borders: an international perspective. The full contents of the supplement are available online at http://www.biomedcentral.com/bmcmededuc/supplements/14/S1. This article was submitted and peer reviewed in 2012. Final acceptance for publication as part of this supplement was in 2014.

Publication of this supplement was supported and funded by the Royal College of Physicians and Surgeons of Canada. The funding agency played no role in the design, in the collection, analysis and interpretation of data; in the writing of the manuscripts; and in the decision to submit the manuscript for publication. The articles have been through the journal's standard peer review process for supplements. The Supplement Editors declare that they have no competing interests.
